# Renal angiomyolipoma with inferior vena cava tumor thrombus in a pregnant woman

**DOI:** 10.1016/j.radcr.2026.04.030

**Published:** 2026-05-07

**Authors:** Chang Shu, Sungmee Park, Diem My Hoang, Dillon Sommer, Medhat Hanna, Roozbeh Houshyar

**Affiliations:** a University of California, San Francisco, School of Medicine - 505 Parnassus Ave, San Francisco, CA 94143, USA; b University of California, Irvine, Computational Abdominal Radiology Lab - 1001 Health Sciences Rd, Irvine, CA 92617, USA; c Johnston Memorial Hospital, 16000 Johnston Mem Dr, Abingdon, VA 24211, USA

**Keywords:** Renal angiomyolipoma, Inferior vena cava, Tumor thrombus, Pregnancy, Vascular invasion, Radical nephrectomy

## Abstract

Renal angiomyolipoma (AML) is a benign mesenchymal tumor that rarely shows aggressive behavior, like vascular invasion. Venous invasion of AML into the inferior vena cava (IVC) is extremely rare, especially in pregnancy, where both diagnosis and treatment are further complicated by maternal and fetal factors. We present the case of a 27-year-old pregnant woman incidentally diagnosed with bilateral renal AMLs, including a right-sided lesion with tumor thrombus extending into the intrahepatic IVC. This diagnosis was made using multiple imaging modalities: ultrasound, magnetic resonance imaging, and postpartum computed tomography. Imaging confirmed macroscopic fat within the tumors and delineated the extent of vascular involvement. Per patient decision, she was closely monitored during pregnancy, then underwent a successful open right radical nephrectomy and IVC thrombectomy postpartum. This case highlights the crucial role of imaging in detecting rare aggressive behavior in an otherwise benign tumor and shows the importance of multidisciplinary planning to prevent more serious complications.

## Introduction

Renal angiomyolipoma (AML) is a common benign solid renal tumor, composed of variable proportions of adipose tissue, dysmorphic blood vessels, and smooth muscle [1]. Approximately 80% of AMLs are sporadic and unilateral, with a strong female predominance, while the remaining 20% are associated with tuberous sclerosis complex (TSC), where they are often bilateral and multiple [2]. These lesions are typically diagnosed by identification of macroscopic intralesional fat on CT and MRI [3].

Although most AMLs follow an indolent course, their primary complication is spontaneous retroperitoneal hemorrhage, particularly for tumors larger than 4 cm [4]. Far more rarely, AMLs may exhibit aggressive features such as invasion into the renal vein or inferior vena cava (IVC), a behavior more commonly found in renal cell carcinoma (RCC) [5]. Vascular invasion carries a risk of pulmonary embolism and often requires major surgical intervention [6]. In pregnancy, AMLs may express estrogen and progesterone receptors; combined with increased gestational vascularity, this can predispose the tumor to rapid growth and intravascular invasion [7]. We report a rare case of renal AML with IVC tumor thrombus in a pregnant woman, emphasizing the central role of imaging in diagnosis and surgical planning.

### Case report

A 27-year-old woman (G2P1) with a history of recurrent urinary tract infections (UTIs) was admitted at 22 weeks of gestation with pyelonephritis. She had no personal or family history of renal tumors, and no clinical signs of TSC or Von Hippel–Lindau syndrome.

**Fig. 1:**
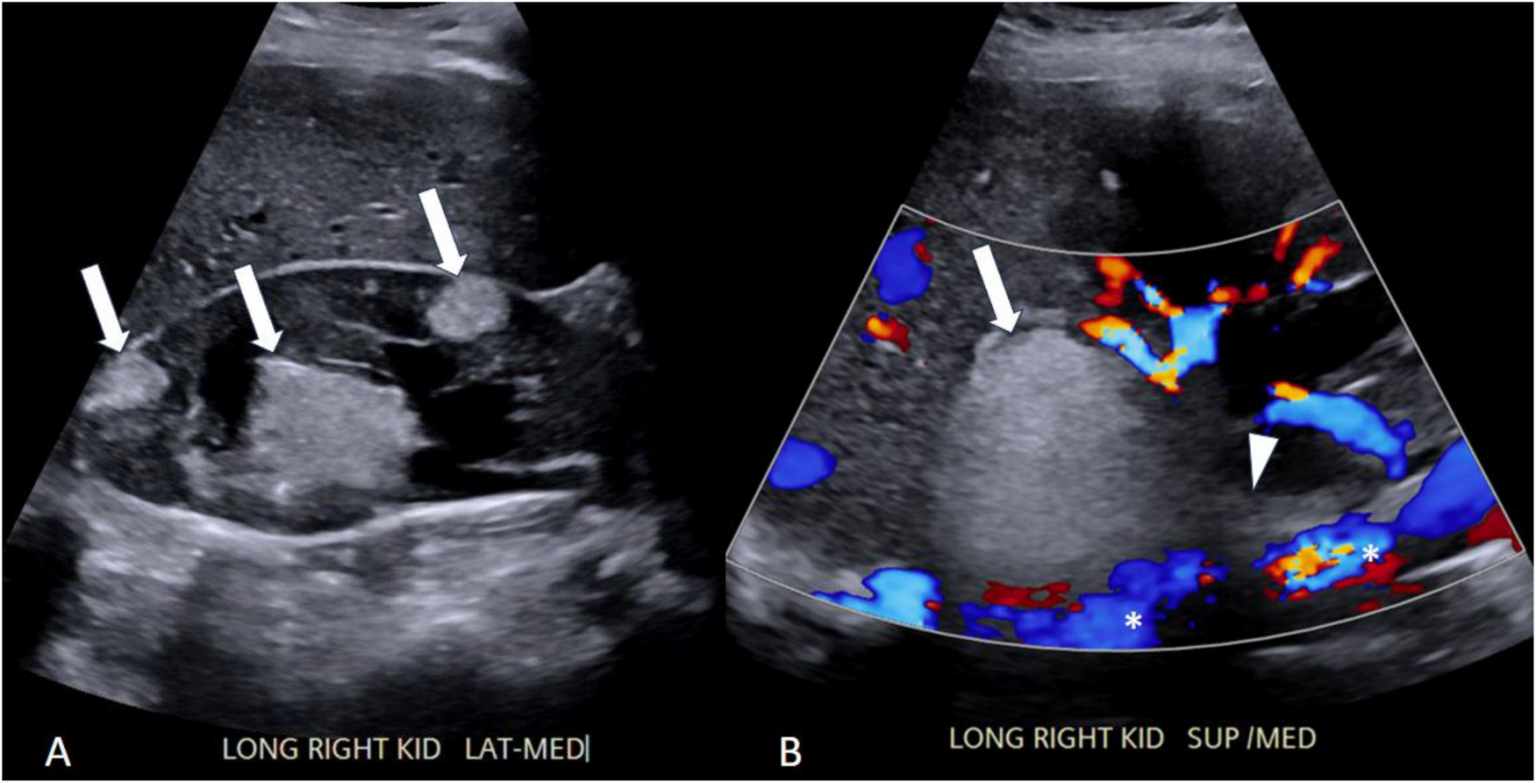
Ultrasound of the right kidney. Grayscale image in the longitudinal plane (A) shows multiple well-circumscribed hyperechoic lesions (white arrows), consistent with fat-containing masses. Color flow doppler image of the right kidney (B) shows no significant blood flow in the lesion (white arrow). The lesion appears to extend medially (arrowhead) to the IVC (*).

Renal US revealed multiple bilateral hyperechoic renal masses consistent with AMLs. Doppler imaging unexpectedly demonstrated extension of echogenic solid material into the IVC (Fig. 1). Subsequent MRI of the abdomen and pelvis without contrast confirmed bilateral renal masses with T1-hyperintensity and T2-signal dropout on fat-saturated sequences, consistent with AMLs; the dominant right upper-pole lesion demonstrated contiguous extension of T2-hypointense material into the right renal vein and IVC. Due to concerns for IVC tumor thrombus and potential maternal risk, a multidisciplinary discussion was held between radiology, urology, and maternal-fetal medicine. Urology determined that the AML was unlikely to invade the renal vein or the IVC, and it was not a potential source for clot formation. The patient was educated on the risk of spontaneous bleeding from the AML and counseled on AML angio-embolization; however, she elected to continue close surveillance and to defer definitive management until postpartum.

**Fig. 2:**
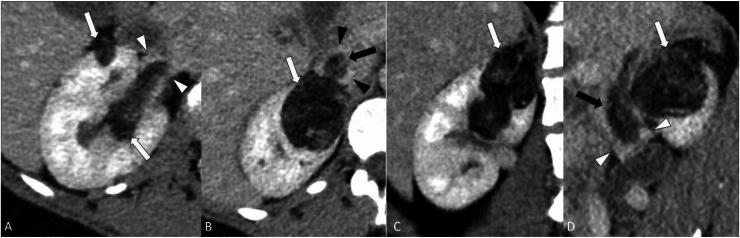
Contrast-enhanced CT of the right kidney demonstrating on axial image (A) several fat-containing renal masses (white arrows) with extension of one lesion into the renal vein (white arrowheads). On a more superior axial image (B) a dominant fat containing renal mass is seen (white arrow) with a corresponding fat containing lesion (black arrow) in the adjacent inferior vena cava (black arrowheads). On the coronal reformatted image (C) the superior-to-inferior extent of the dominant lesion is again seen (white arrow). On the sagittal view (D) the dominant lesion is present (white arrow) with extension of fat-density material (black arrow) into the right renal vein and IVC (white arrowheads).

Following delivery, CT with contrast confirmed multiple bilateral fat-containing renal tumors. The dominant right upper-pole mass measured 3.9 × 3.3 cm and extended through the right renal vein into the intrahepatic IVC (Fig. 2). The patient underwent an open right radical nephrectomy with IVC tumor thrombectomy and caval reconstruction. Her recovery was uneventful, and she remains under surveillance for the contralateral renal tumors.

**Fig. 3:**
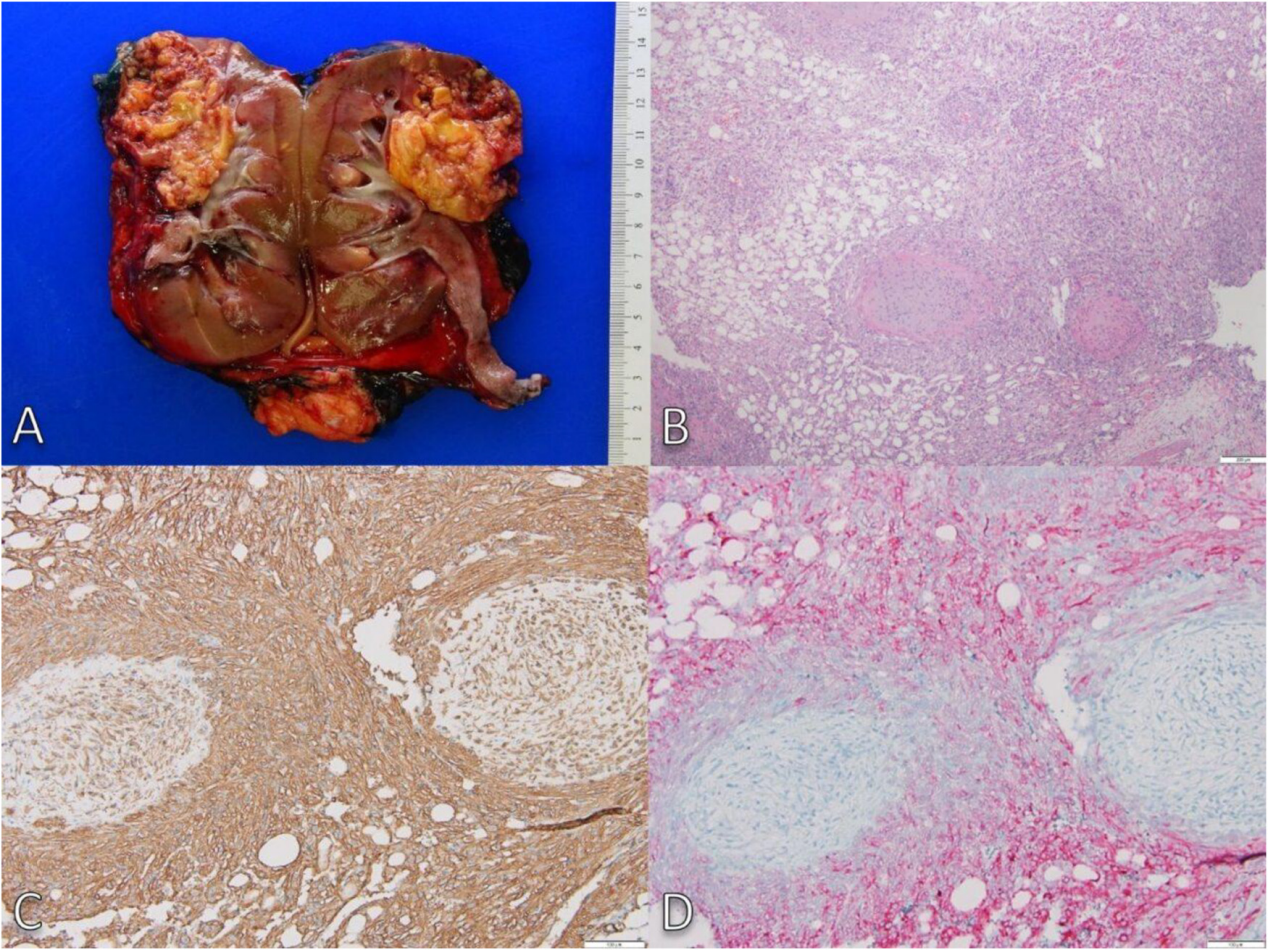
(A) A 4.8 cm AML involving the upper pole of the kidney. The cut surface is predominantly yellow due to mature adipose tissue, with red areas corresponding to vascular components. The tumor infiltrates adjacent renal parenchyma abuts the renal pelvis and involves the renal vein with extension into the inferior vena cava. (B) Typical renal AML composed of adipose tissue, blood vessels, and smooth muscle. The vessels are dysmorphic, with thick walls, irregular lumens, and absent elastic lamina. The smooth muscle component is closely associated with and radiates from vessel walls. (C) Smooth muscle actin (SMA) immunostaining highlights the smooth muscle component and its distribution. (D) Expression of melanocytic markers (HMB45) in myoid (smooth muscle) and adipose components confirm the diagnosis.

On pathology (Fig. 3), the gross examination showed a 4.8 cm AML in the right upper pole. Microscopy confirmed the triphasic composition of adipose tissue, thick-walled vessels, and smooth muscle cells. Immunohistochemistry was positive for smooth muscle actin (SMA) and melanocytic marker HMB-45, confirming AML. Genetic testing for TSC1 and TSC2 mutations was negative. The imaging features of macroscopic fat with a contiguous intravascular soft-tissue component corresponded pathologically to classic triphasic AML tissue extending into the renal vein and IVC lumen, confirming that the IVC filling defect represented true tumor thrombus rather than bland clot. The contralateral AMLs are being monitored with periodic MRI or CT surveillance given their size and absence of vascular invasion.

## Discussion

This case demonstrates an exceptionally rare manifestation of a common benign tumor. While AML is classically indolent, vascular invasion with IVC tumor thrombus represents an aggressive variant with serious potential complications, including pulmonary embolism [8]. The differential diagnosis for fat-containing renal masses includes retroperitoneal liposarcoma and fat-containing RCC, which is also a rare entity [9]. While this behavior is more typical of RCC, the presence of macroscopic fat contiguous with the thrombus favored an aggressive variant of AML, as fat-containing RCC typically presents with only minimal microscopic fat and frequent calcifications.

Imaging played a key role throughout the diagnostic process. US with Doppler first raised suspicion for vascular invasion, while CT provided definitive characterization by identifying macroscopic fat and precisely mapping the thrombus extent. This was essential for surgical planning and risk stratification [5,10,11]. Management ranges from observation or selective arterial embolization for small lesions to radical nephrectomy and thrombectomy when vascular invasion is present [12].

In pregnancy, a multidisciplinary approach is required to balance surgical risks with fetal maturity, often deferring definitive intervention until the postpartum period [13]. The hormonal changes, including elevated estrogen and progesterone levels, may contribute to AML growth and vascular proliferation, potentially predisposing to intravascular invasion [[14], [15], [16]]. Although few cases of AML with IVC involvement in pregnancy have been reported and direct causation of vascular invasion remains speculative, this case adds to the growing evidence that even benign renal neoplasms can demonstrate aggressive clinical behavior.

For radiologists, the key teaching point is to always assess for venous invasion, even when the renal lesion shows classic imaging features of AML. The identification of a tumor thrombus transforms management from conservative observation or embolization [17] to definitive surgical resection, underscoring the importance of meticulous imaging evaluation.

## Conclusion

This case illustrates the rare but clinically significant occurrence of renal angiomyolipoma with direct tumor invasion into the renal vein and IVC in a pregnant patient. Even when a renal tumor has classic benign features, assessment for vascular invasion is critical, as it fundamentally alters management strategy and prognosis. Imaging plays a critical role in detection, staging, and coordinating care across specialties, helping ensure timely treatment and prevent life-threatening complications.

## Ethics approval and consent to participate

This study was conducted in accordance with and approved by the Institutional Review Board (IRB) at University of California, Irvine.

## Patient consent

Consent was obtained from the patient for publication of this case report and any accompanying images.
